# Circular RNA hsa_circ_0000326 acts as a miR-338-3p sponge to facilitate lung adenocarcinoma progression

**DOI:** 10.1186/s13046-020-01556-4

**Published:** 2020-04-05

**Authors:** Yuzhu Xu, Jun Yu, Zhenli Huang, Bohua Fu, Yu Tao, Xuefei Qi, Yong Mou, Yinan Hu, Yi Wang, Yong Cao, Dingsheng Jiang, Jungang Xie, Yongjian Xu, Jianping Zhao, Weining Xiong

**Affiliations:** 1 Department of Respiratory and Critical Care Medicine, Wuhan Clinical Medical Research Center for Chronic Airway Medicine, NHC Key Laboratory of Pulmonary Diseases, Key cite of National Clinical Research Center for Respiratory Diseases, Tongji Hospital, Tongji Medical College, Huazhong University of Sciences & Technology, 1095 Jiefang Ave, Wuhan, 430030 China; 2 Department of Respiratory, Zhuzhou Central Hospital, Zhuzhou, China; 3 Department of Thoracic Surgery, Tongji Hospital, Tongji Medical College, Huazhong University of Science and Technology, Wuhan, China; 4 Division of Cardiothoracic and Vascular Surgery, Tongji Hospital, Tongji Medical College, Huazhong University of Science and Technology, Wuhan, China; 5 Department of Respiratory Medicine, Shanghai Ninth People’s Hospital, Shanghai Jiaotong University School of Medicine, 639 Zhizaoju Lu, Shanghai, 200011 China

## Abstract

**Background:**

Circular RNAs (circRNAs) are a novel class of noncoding RNAs that regulate gene expression at the transcriptional or posttranscriptional level. According to recent studies, circRNAs are involved in the pathogenesis of cancer, but the roles of circRNAs in lung adenocarcinoma are largely unknown.

**Methods:**

In this study, we identified a novel upregulated circRNA, hsa_circ_0000326, in human lung adenocarcinoma tissues using microarray analysis and qRT-PCR. We then explored the biological role of hsa_circ_0000326 using gain- and loss-of-function assays in adenocarcinoma cells. Bioinformatics databases were used to screen for potential target miRNAs and the luciferase reporter assays and RNA-FISH further validated the interaction. Downstream protein was detected by western blot. Finally, we established xenografts in nude mice to assess the function of hsa_circ_0000326 in vivo.

**Results:**

We found that high expression of hsa_circ_0000326 was correlated with tumor size, regional lymph node status and differentiation in human lung adenocarcinoma. Additionally, we conducted gain- and loss-of-function assays and found that hsa_circ_0000326 acted as a positive regulator of cell proliferation and migration and a negative regulator of apoptosis. Mechanistic studies showed that hsa_circ_0000326 acted as a miR-338-3p sponge and altered the function of miR-338-3p, which in turn upregulated the expression of the downstream target RAB14 and affected the proliferation, migration and apoptosis of lung adenocarcinoma cells.

**Conclusions:**

Collectively, our study results reveal crucial roles for hsa_circ_0000326 in the proliferation, migration and apoptosis of lung adenocarcinoma cells and suggest that hsa_circ_0000326 may represent a potential therapeutic target in patients with lung adenocarcinoma.

## Introduction

Lung cancer is one of the most common causes of cancer-related death [1], and the 5-year survival rate for patients with lung cancer is only 16.8% [2]. Non-small cell lung cancers (NSCLCs) account for 85% of all cases [3], among which lung adenocarcinoma is the most common histological type. Therefore, molecular studies aimed at identifying promising targets for the treatment of lung adenocarcinoma are urgently needed.

Circular RNAs (circRNAs) are produced by the circularization of exons, exons and introns, or intron sequences alone and are widely expressed in various cell types [4, 5]. Since circRNAs usually do not display protein-coding potential, they were once considered simply to be accidental byproducts of splicing [6]. Advances in RNA sequencing technologies have accelerated the identification of circRNAs. CircRNAs are abundant and conserved RNA isoforms that are more stable than linear RNAs [7, 8]. Divergent primers (primers in a primer pair that face away from each other) that cross over back-splicing sites have been used to amplify particular circular transcripts [9]. CircRNAs have also been recognized as miRNA sponges and transcriptional regulators [9–11]. Moreover, they have been found to interact with RNA-binding proteins or be translated into proteins in vitro [12–14]. Further studies on circRNAs might provide new insights that will improve our understanding of pathological mechanisms and improve the prevention and diagnosis of related diseases. Previous studies have shown that circRNAs might be involved in the pathogenesis of lung cancer [15, 16]. However, the exact roles of circRNAs in lung adenocarcinoma are far from known.

Hsa_circ_0000326 is located on chromosome 11:65272490–65,272,586, and its associated gene symbol is MALAT1. Interestingly, in our early screening experiment, hsa_circ_0000326 was found to be markedly upregulated in a cohort of lung adenocarcinoma tissues and adjacent tissues by microarray analysis. Herein, we provide evidence that aberrant hsa_circ_0000326 expression could promote proliferation and migration and inhibit apoptosis in lung adenocarcinoma cells. Further study showed that hsa_circ_0000326 acted as a miR-338-3p sponge to inhibit its activity and thus upregulate its target RAB14, which in turn affected proliferation, migration and apoptosis in lung adenocarcinoma cells. Based on these results from the present study, targeting hsa_circ_0000326 may be a viable treatment strategy for lung adenocarcinoma.

## Materials and methods

### Ethics statement and patient samples

Human lung adenocarcinoma tissues and corresponding adjacent tissues (> 5 cm from the tumor edge) (*N* = 100) were obtained from patients who received surgical treatment at Tongji Hospital (Wuhan, China) from June 2014 to February 2015. Fresh tissues were immediately snap-frozen and stored at − 80 °C. All of the patients gave written informed consent, and none of the patients had previously undergone radiotherapy or chemotherapy. This study was approved by the Human Assurance Committee of Tongji Hospital, Tongji Medical College, Huazhong University of Science and Technology.

### CircRNA microarray

Five pairs of lung adenocarcinoma and adjacent tissues were used to detect the expression of circRNAs with a CircRNA Microarray (KangChen Biotech, Shanghai, China). Sample preparation and microarray hybridization were performed according to standard protocols from Arraystar (Rockville, MD, USA). Briefly, total RNA from each sample was amplified and transcribed into fluorescent cRNA using random primers according to the Arraystar Super RNA Labeling Kit protocol. Labeled cRNAs were hybridized onto an Arraystar Human circRNA Array (Arraystar Inc., MD, USA). After the slides were washed, the arrays were scanned using an Axon GenePix 4000B microarray scanner. The scanned images were then imported into GenePix Pro 6.0 software for grid alignment and data extraction. Quantile normalization and subsequent data processing were performed using the R software package. All of the circRNAs array data were listed in Supplement Table 1.

### Cell culture

Human lung adenocarcinoma cell lines (A549, SPC-A1, H1299 and H1975) were purchased from the Type Culture Collection of the Chinese Academy of Sciences (Shanghai, China). And the cell lines was authenticated by short tandem repeat method at Suzhou Genetic Testing Biotechnology Co.,Led. (Suzhou, China) in Feb., 2018. The cells were cultured in Roswell Park Memorial Institute (RPMI) 1640 medium supplemented with 10% fetal bovine serum (FBS), 100 mg/mL streptomycin and 100 IU/mL penicillin in a 5% CO_2_ .

### Cell transfection

Three siRNAs targeting hsa_circ_0000326 and a nontargeting control (named si-circ0000326–1, si-circ0000326–2, si-circ0000326–3 and si-NC, respectively), miRNA mimics, a miRNA inhibitor and a negative control miRNA (miRNA-NC) were obtained from RiboBio (Guangzhou, China). The sequence for siRNAs were as follows: si-circ0000326–1: 5′-GTAACTGGCATGTGAACAA-3′; si-circ0000326–2: 5′-ACTGGCATGTGAACAAGCT-3′; si-circ0000326–3:5′-TGGCATGTGAACAAGCTTT-3′. The sequence of mature hsa_circ_0000326 was synthesized and inserted into a circRNA vector plasmid (named pAV-circ0000326) from Vigene Biosciences (Rockville, MD, USA). The transfection of siRNAs and plasmids was optimized using Lipofectamine 3000 (Invitrogen, Carlsbad, CA, USA), according to the manufacturer’s instructions.

### Generation of stable cell lines

The si-circ0000326 or si-NC sequence was inserted into the lentiviral expression vector piLenti-siRNA-GFP and packaged into viral particles (LV-si-circ0000326 or LV-NC, respectively). H1299 cells were infected with either LV-si-circ0000326 or LV-NC particles. Three days after infection, the cells were selected in medium containing 5 μg/mL puromycin for 7 days and then maintained in medium containing 1 μg/mL puromycin.

### RNA extraction and RT-PCR

Total RNA was isolated using TRIzol reagent (TaKaRa, Tokyo, Japan) as previously described [17, 18]. For quantitative PCR analysis of circRNAs and mRNAs, total RNA (500 ng) from each sample was transcribed into first-strand cDNA using a Prime Script™ RT Master Mix kit (TaKaRa, Tokyo, Japan). Real-time PCR was performed using SYBR® Premix Ex Taq™ with an ABI Prism 7500 detection system according to the manufacturers’ instructions. The data were analyzed using the 2^-ΔΔCt^ method and normalized to β-actin expression levels. The primers for human hsa_circ_0000326 and β-actin were as follows: hsa_circ_0000326, forward: 5′-TTG AAT AGA TTT CAG CTT TAT GC-3′ and reverse: 5′-CCC ATA ACT GAT CTG ACT TTG T-3′; β-actin, forward: 5′-CCT GGC ACC CAG CAC AAT-3′ and reverse: 5′-GGG CCG GAC TCG TCA TAC-3′.

To detect the miRNA expression level, a total of 1 μg miRNAs were reverse transcripted with Bulge-LoopTM miRNA qRT-PCR Primer Set (Ribobio, Guangzhou, China) and the miR-338-3p expression was detected with SYBR® Premix Ex Taq™ (TaKaRa, Tokyo, Japan). Primers for miR-338-3p and U6 snRNA were purchased from Ribobio.

### Flow cytometry analysis

For cell cycle assays, transfected cells were harvested, diluted to a density of 1 × 10^6^ cells/mL and fixed with 70% ice-cold ethanol. Next, the cells were stained with 400 μL of a propidium iodide solution (Keygen Biotech, Nanjing, China) for 30 min and then subjected to cell cycle analysis using flow cytometry (BD Biosciences, San Jose, CA, USA).

For apoptosis analysis, 300 nM H_2_O_2_ was applied to induce apoptosis. Cells were collected 48 h after transfection and resuspended in binding buffer. The cells were then incubated with annexin V and propidium iodide (Keygen Biotech, Nanjing, China) for 15 min in the dark and then analyzed by flow cytometry on a FACSCalibur flow cytometer.

### Cell proliferation assay

Transfected cells were seeded in a 96-well plate at a density of 4000 cells per well and cultured for 24 h, 48 h, 72 h or 96 h. Cell Counting Kit 8 (CCK-8) solution (Keygen Biotech, Nanjing, China) was added to each well, and the cells were then incubated at 37 °C for 60 min. The absorbance was measured at 450 nm with a spectrophotometer. The data are representative of three individual experiments performed in triplicate.

For colony formation assays, 450 transfected cells were seeded in each well of a 6-well plate and maintained for 10 days, with replacement of the medium every 3 days. The colonies were then fixed with methanol, stained with 0.5% crystal violet, and counted under a microscope.

### Transwell assay

For the migration assay, 2 × 10^4^ transfected cells were suspended in 200 μL of serum-free medium and then seeded in the upper chamber of each transwell insert (Corning, NY, USA); 600 μL of culture medium containing 15% FBS was added to the lower chamber.

After 24 h of incubation, the cells that had migrated were fixed with 4% paraformaldehyde and stained with 0.5% crystal violet, and the cells remaining on the upper surface of the filter membrane were removed with a cotton swab. Images of cells on the lower surface of the membrane were captured using a microscope equipped with a camera.

### Western blot analysis

Total proteins were extracted using RIPA lysis buffer supplemented with a protease inhibitor cocktail, as previously described [19, 20]. Proteins were separated by SDS-PAGE and then blotted onto PVDF membranes. The membranes were incubated with primary antibodies overnight at 4 °C and then incubated with secondary antibodies for 1 h at room temperature. Finally, the proteins were detected by chemiluminescence according to the manufacturer’s recommendations (Advansta, CA, USA). The following primary antibodies were used: anti-RAB14 (1:1000, Proteintech, Wuhan, China), anti-ZEB2 (1:1000, Proteintech, Wuhan, China), anti-SOX4 (1:1000, Millipore, Billerica, MA, USA), and anti-GAPDH (1:2000, Sungene, Tianjin, China).

### Bioinformatics analysis and luciferase reporter assay

The relationships between circRNAs and miRNAs were predicted with custom Arraystar miRNA target prediction software based on miRanda (http://www.microrna.org) and TargetScan (www.targetscan.org). The target genes of miRNAs were acquired from TargetScan, PicTar and miRanda. The putative binding site of hsa_circ_0000326 or putative binding site mutants were inserted into the MCS of the pmirGLO vector for use in a dual luciferase reporter assay (abm, Richmond, Canada). HEK293T cells were cotransfected with wild-type or mutant pmirGLO-hsa_circ_0000326 (WTpmirGLOcirc0000326 or MTpmirGLOcirc0000326, respectively) along with 50 nM miR-NC, miR-338-3p, miR-9-3p, miR-16-2-3p, miR-320a or miR-320b mimic using Lipofectamine 2000 (Life Technologies, MA, USA). Firefly and Renilla luciferase activity was measured with a Dual-Luciferase Reporter System (Promega, Madison, WI, USA) 48 h after transfection. The effect of each miRNA on the activity of the luciferase reporter containing the hsa_circ_0000326 sequence was normalized to the activity of the luciferase reporter cotransfected with miRNA-NC.

### Fluorescence in situ hybridization (FISH)

RNA-FISH was used to detect the subcellular localization of hsa_circ_0000326 and miR-338-3p. Cells were grown on cover slips, fixed with 4% paraformaldehyde for 20 min, incubated with proteinase K and washed with a series of alcohol solutions. Then, the slides were washed and incubated with prehybridization solution (BersinBio, Guangzhou, China) for 30 min at 37 °C. FAM-labeled hsa_circ_0000326 probes (5′-AAG CTT GTT CAC ATG CCA GTT ACT-3′) and Cy3-labeled miR-338-3p probes (5′-CAA CAA AAT CAC TGA TGC TGG A-3′) (Sangon Biotech, Shanghai, China) were denatured at 73 °C for 8 min and hybridized to the slides for 24 h at 42 °C. Subsequently, blocking was performed, and 4,6-diamidino-2-phenyl-indole (DAPI) was used to stain the cell nuclei. Images were obtained with a confocal microscope (Olympus, Shinjuku, Japan).

### RNA pulldown assay

RNA pulldown assay was conducted by Shanghai Ruantuo Biological Technology Co. (Shanghai, China) using established techniques [21].

### Establishment of tumor xenografts in nude mice

Male BALB/c nude mice (4–5 weeks of age) were purchased from Hunan SJA Laboratory Animal Company (Changsha, China) and maintained under specific pathogen-free conditions. Cells stably infected with LV-si-circ0000326 or LV-NC H1299 (1 × 10^6^ cells in 100 μL of PBS) were subcutaneously injected into the right armpits of nude mice. The tumor size was measured every 7 days. Three weeks later, the mice were sacrificed, and the weight of each tumor was measured.

### Statistical analysis

All experiments were performed in triplicate. Continuous data are presented as the mean ± standard deviation (SD). Paired t tests, independent t tests, chi-square tests and one-way analysis of variance (ANOVA) were used in this study as appropriate. GraphPad Prism 5.0 software (GraphPad Software Inc., San Diego, CA, USA) and SPSS 22.0 software (IBM Corp., Armonk, NY, USA) were used for statistical analysis of the data. A two-tailed *P*-value < 0.05 was considered to indicate a statistically significant difference.

## Results

### Hsa_circ_0000326 expression was upregulated in lung adenocarcinoma tissues and associated with clinical characteristics

**Fig. 1:**
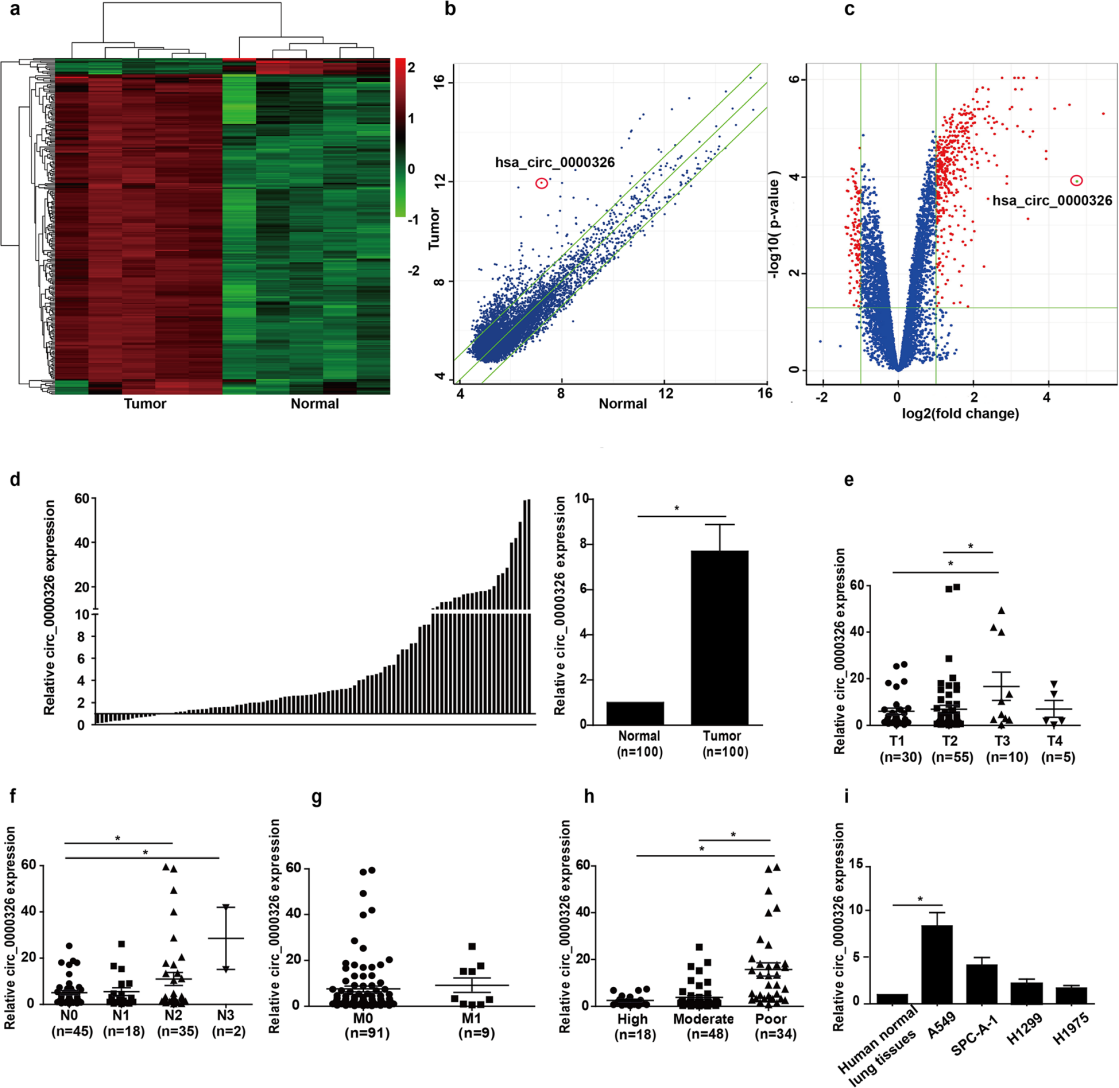
Upregulation of hsa_circ_0000326 was correlated with aggressive characteristics of lung adenocarcinoma. (**a**) Hierarchical clustering showing different circRNA expression profiles between lung adenocarcinoma tissues and normal tissues. (**b**) Scatter plots showing the circRNA expression variation between the two compared groups. (**c**) Volcano plots were used to visualize differential expression between the two different conditions. (**d**) Expression of hsa_circ_0000326 in the 100 paired human lung adenocarcinoma tissues and adjacent normal tissues. (**e**) Correlation between hsa_circ_0000326 expression and T classifications (T1-T4) in 100 cases of lung adenocarcinoma tissues and normal lung tissues. (**f**) Correlation between hsa_circ_0000326 expression and N classifications of lung adenocarcinoma (N0-N3). (**g**) Correlation between hsa_circ_0000326 expression and differentiation of lung adenocarcinoma. (**h**) Correlation between hsa_circ_0000326 expression and distant metastasis. (**i**) Relative hsa_circ_0000326 expression levels in the four lung adenocarcinoma cell lines. *P < 0.05

A total of 8247 circRNAs were detected by microarray analysis in five pairs of lung adenocarcinoma and adjacent noncancerous tissues. Hierarchical clustering revealed that different circRNA expression patterns between cancer and adjacent noncancerous tissues (Fig. 1a). Scatter plots and volcano plots were used to visualize the differentially expressed circRNAs that showed statistically significant differences between tissue types (Fig. 1b and c). The expression of 568 circRNAs was altered in lung adenocarcinoma tissues, among which 448 circRNAs were upregulated and 120 circRNAs were downregulated (fold change≥2.0, *P* < 0.05 and FDR < 0.05). The top ten upregulated and downregulated circRNAs are listed in Supplement Table 2. Consistent with the data from the microarray analysis, hsa_circ_0000326 was found to be markedly upregulated in an expanded cohort of lung adenocarcinoma tissues and adjacent tissues (*N* = 100) by RT-PCR (Fig. 1d).

**Fig. 2:**
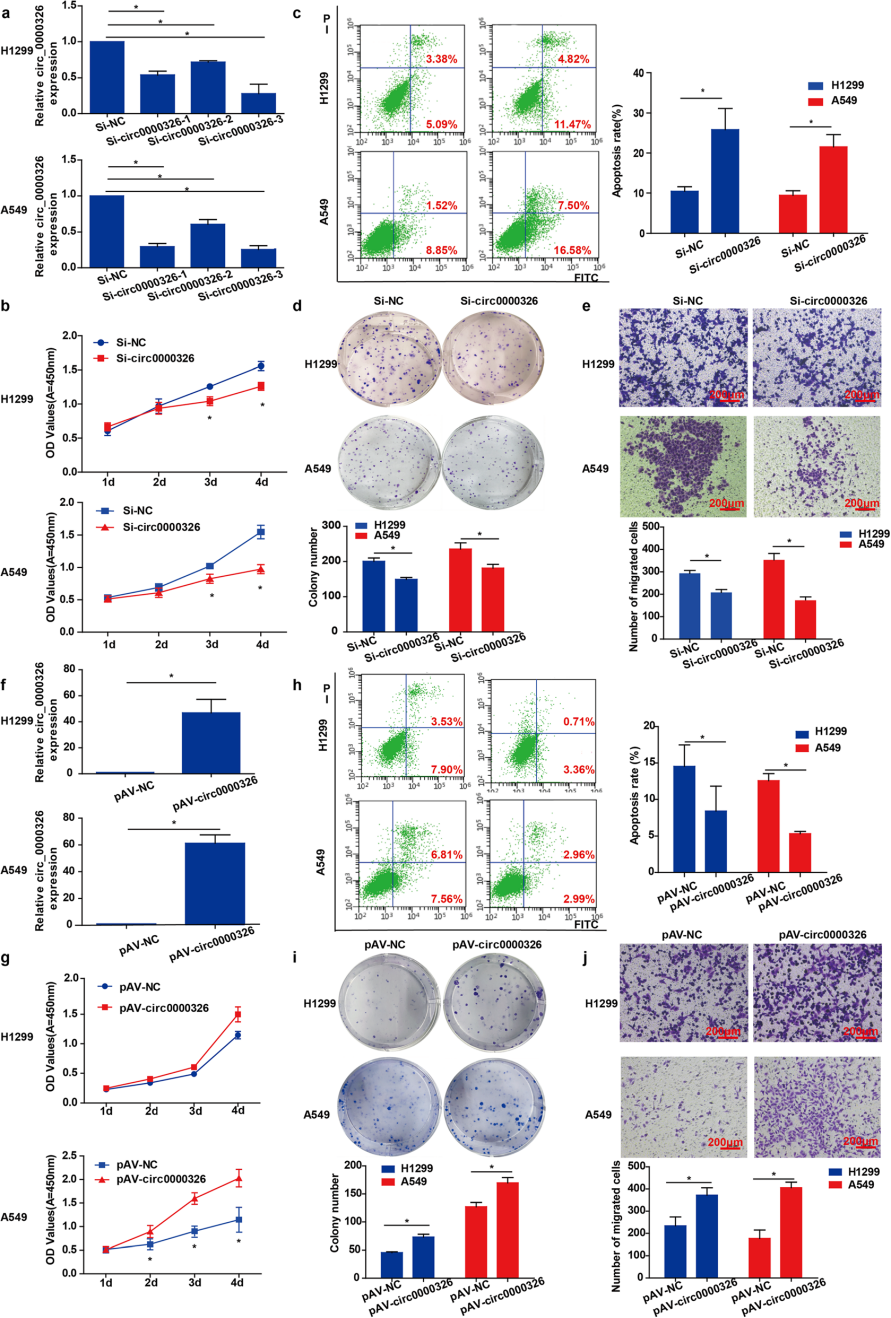
Effects of hsa_circ_0000326 expression on proliferation, apoptosis and migration in lung adenocarcinoma cells. (**a**) Hsa_circ_0000326 expression levels were determined by RT-PCR. (**b**) Effect of hsa_circ_0000326 knockdown on cell proliferation, as determined by CCK-8 assay. (**c**) Effect of hsa_circ_0000326 knockdown on cell apoptosis. (**d**) Effect of hsa_circ_0000326 knockdown on cell proliferation, as determined by colony formation assay. (**e**) Effect of hsa_circ_0000326 knockdown on cell migration. (**f**) Hsa_circ_0000326 expression levels were determined by RT-PCR. (**g**) Effect of hsa_circ_0000326 overexpression on cell proliferation, as determined by CCK-8 assay. (**h**) Effect of hsa_circ_0000326 overexpression on apoptosis. (**i**) Effect of hsa_circ_0000326 overexpression on cell proliferation, as determined by colony formation assay. (**j**) Effect of hsa_circ_0000326 overexpression on cell migration. Magnification: 100×. Scale bar = 200 μm. The bars with error bars represent the mean ± SD from three independent experiments. *P < 0.05

Next, correlations between hsa_circ_0000326 expression and patient clinical pathological parameters were analyzed, and the results revealed that hsa_circ_0000326 expression was significantly correlated with tumor size (T1 vs T3, *P* = 0.0143; T2 vs T3, *P* = 0.0366, Fig. 1e), lymphatic metastasis (*P* = 0.004, Fig. 1f) and tumor differentiation stage (*P* < 0.001, Fig. 1g) in patients with lung adenocarcinoma. However, high expression of hsa_circ_0000326 was not correlated with patient age (*P* = 0.797), sex (*P* = 0.258), smoking status (*P* = 0.181) or distant metastasis (*P* = 0.537, Fig. 2g) (Supplement Table 3). These results suggested a link between upregulation of hsa_circ_0000326 and aggressive characteristics of lung adenocarcinoma.

### Hsa_circ_0000326 promoted proliferation and migration and inhibited apoptosis in lung adenocarcinoma cells

To further explore the role of hsa_circ_0000326 in the progression of lung adenocarcinoma, we performed loss-of-function and gain-of-function assays. Based on the levels of endogenous hsa_circ_0000326 expression in the four lung adenocarcinoma cell lines (Fig. 1i), the A549 and H1299 cell lines were chosen as our experimental cell lines. Specifically, designed siRNAs targeting hsa_circ_0000326 and si-NC were used to transfect cells. Hsa_circ_0000326 expression levels were reduced to the greatest extent in cells transfected with si-circ0000326–3, which was thus used in subsequent analysis (Fig. 2a). In contrast, an hsa_circ_0000326 overexpression cell model was generated by transfecting an hsa_circ_0000326 expression plasmid into A549 or H1299 cells. RT-PCR was used to verify the upregulation of hsa_circ_0000326 in cells transfected with pAV-circ0000326 compared to those transfected with the negative control plasmid (pAV-NC) (Fig. 2f). Cell proliferation was assessed by CCK-8 assay. Silencing of hsa_circ_0000326 expression suppressed the proliferation of A549 and H1299 cells (Fig. 2b), while overexpression of hsa_circ_0000326 significantly increased the proliferation of A549, and a similar trend was also observed in H1299 cells (Fig. 2g). Similarly, based on the results of a colony formation assay, hsa_circ_0000326 knockdown significantly inhibited colony formation (Fig. 2d), while hsa_circ_0000326 overexpression promoted colony formation (Fig. 2i). Flow cytometry analysis was used to investigate the effects of hsa_circ_0000326 on the cell cycle and apoptosis and to explore the possible effect of hsa_circ_0000326 on proliferation. Cells transfected with si-circ0000326 or an overexpression plasmid showed no significant changes in the percentages of cells in the G0/G1, S, or G2/M phases (Supplement Fig. 1A-B). However, apoptosis analysis revealed knockdown of hsa_circ_0000326 expression promoted apoptosis in A549 and H1299 cells (Fig. 2c), while significantly lower apoptosis rates were observed in hsa_circ_0000326-overexpressing cells (Fig. 2h). Thus, hsa_circ_0000326 facilitated lung adenocarcinoma cell proliferation partially by inhibiting apoptosis rather than by affecting the cell cycle.

To identify the role of hsa_circ_0000326 in lung adenocarcinoma metastasis, transwell insert chambers were used to investigate the impact of hsa_circ_0000326 on cell migration, as measured by crystal violet staining. Compared to the control treatment, silencing of hsa_circ_0000326 suppressed the migration of A549 and H1299 cells across the transwell membrane (Fig. 2e). Conversely, hsa_circ_0000326 overexpression prominently increased A549 and H1299 cell migration (Fig. 2j).

Collectively, the data revealed that hsa_circ_0000326 enhanced proliferation and migration and inhibited apoptosis in lung adenocarcinoma cells.

### Hsa_circ_0000326 functioned as a miR-338-3p sponge and interacted with miR-338-3p

**Fig. 3:**
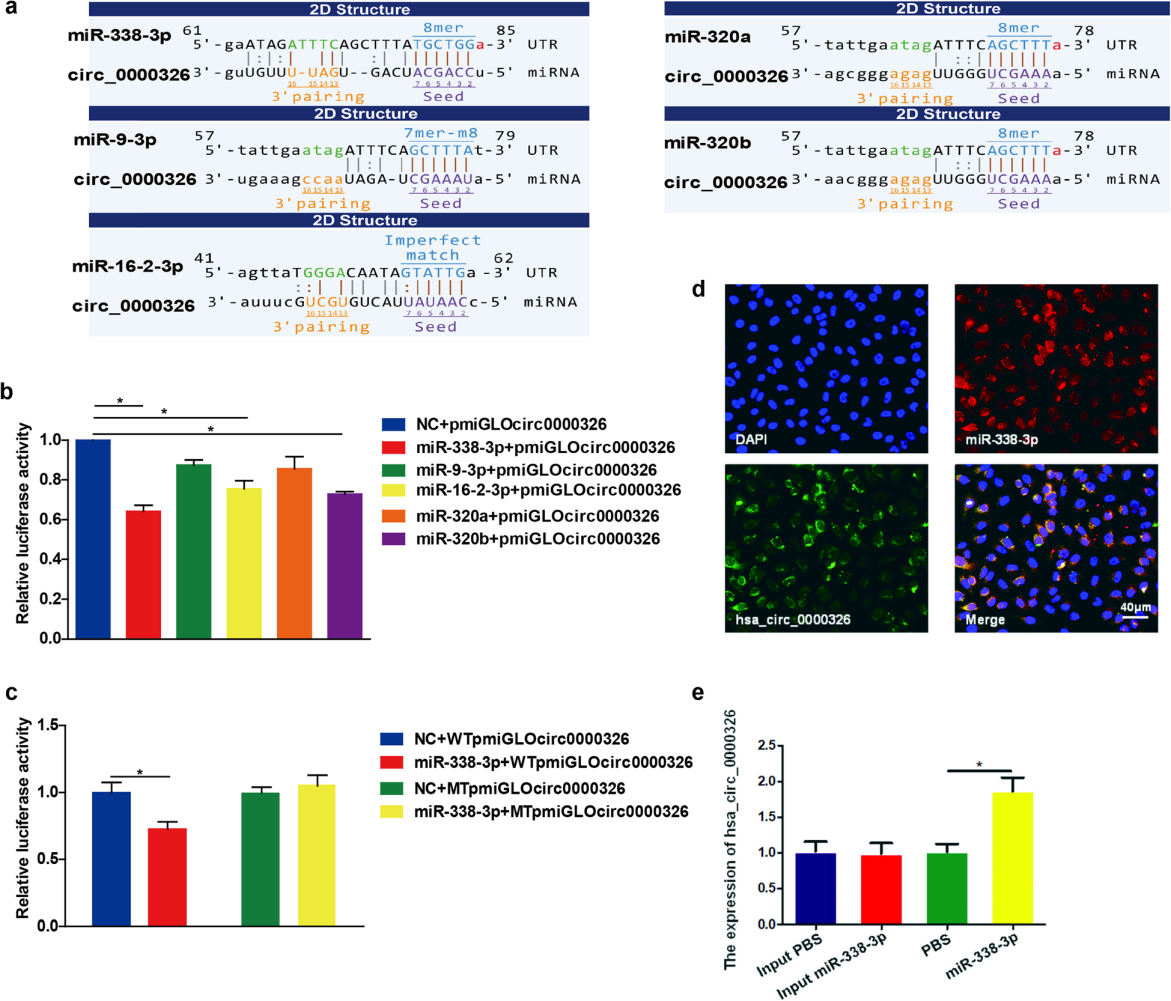
Hsa_circ_0000326 acted as a sponge of miR-338-3p. (**a**) Putative targets of hsa_circ_0000326 were predicted using custom Arraystar software. (**b**) Effects of five predicted miRNAs and a negative control on the luciferase activity of hsa_circ_0000326, as determined by luciferase reporter assay. (**c**) The results of the luciferase reporter assay validated the interaction between hsa_circ_0000326 and miR-338-3p. (**d**) Colocalization between hsa_circ_0000326 and miR-338-3p was observed (arrowheads) using RNA-FISH in A549 cells. The nuclei were stained with DAPI. Scale bar = 40 μm. (**e**) RNA pulldown assay in A549 cells showed hsa_circ_0000326 could interact with miR-338-3p. The bars with error bars represent the mean ± SD from three independent experiments. *P < 0.05

Based on accumulating evidence, circRNAs possess numerous binding sites for miRNAs and can function as miRNA sponges to inhibit the activation of miRNAs; such sponging is one of the most common functions of circRNAs [22–25]. Thus, we hypothesized that hsa_circ_0000326 might serve as a miRNA sponge. A bioinformatics database was used to screen for miRNAs that might contain binding sites for hsa_circ_0000326. We filtered the results by condition, and five target miRNAs (miR-338-3p, miR-9-3p, miR-16-2-3p, miR-320a, and miR-320b) were chosen for further validation (Fig. 3a). To assess whether hsa_circ_0000326 could indeed bind to the predicted miRNAs, a dual luciferase reporter assay was performed. Luciferase-hsa_circ_0000326 reporters were cotransfected with miRNA mimics or negative controls, and the results indicated that compared with miRNA-NC, miR-338-3p reduced luciferase activity by 36% (Fig. 3b). Next, WTpmirGLOcirc0000326 or MTpmirGLOcirc0000326 plasmids were transiently cotransfected into cells with miR-338-3p mimics; luciferase activity was markedly lower in the wild-type hsa_circ_0000326 vector group than in the mutant vector group (Fig. 3c). Therefore, hsa_circ_0000326 interacted with miR-338-3p. Additionally, the subcellular localization of hsa_circ_0000326 and miR-338-3p was examined using RNA-FISH. Notably, hsa_circ_0000326 was mainly localized in the cytoplasm of A549 cells, consistent with the localization of miR-338-3p (Fig. 3d). In the figure, the merged picture shows the colocalization of hsa_circ_0000326 and miR-338-3p. Furthermore, RNA pulldown assay was conducted and showed the similar result (Fig. 3e). Based on these data, hsa_circ_0000326 could directly bind with miR-338-3p in the cytoplasm.

### The effects of hsa_circ_0000326 on lung adenocarcinoma cells depended on miR-338-3p

**Fig. 4:**
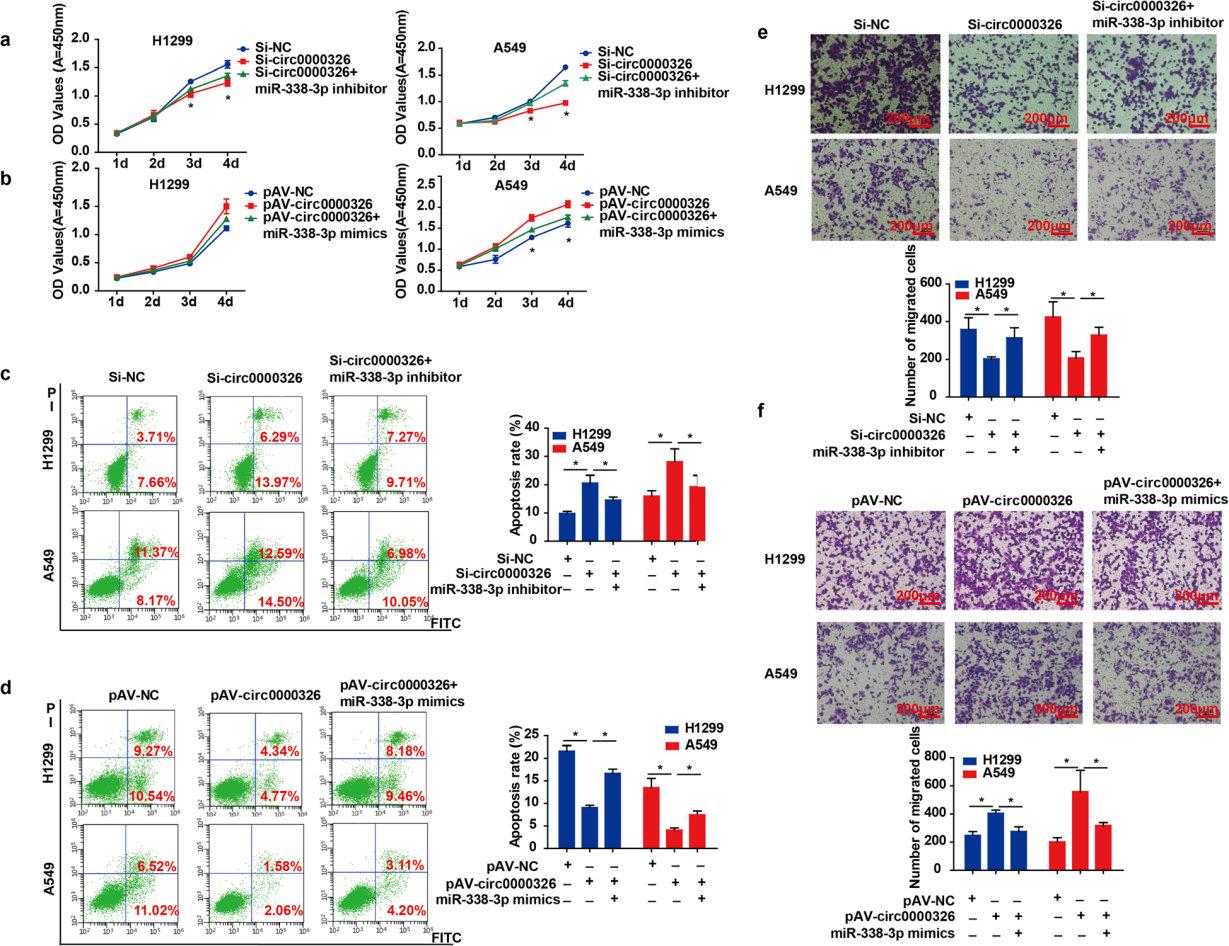
Hsa_circ_0000326 promoted proliferation and inhibited apoptosis in lung adenocarcinoma cells by regulating miR-338-3p. (**a**, **b**) Effect of miR-338-3p on hsa_circ_0000326-induced cell proliferation, as determined by CCK-8 assay. (**c**, **d**) Effect of miR-338-3p on hsa_circ_0000326-induced apoptosis. (**e**, **f**) Effect of miR-338-3p on hsa_circ_0000326-induced cell migration, as determined by Boyden chamber assay. The bars with error bars represent the mean ± SD from three independent experiments. *P < 0.05

We next examined whether miR-338-3p was responsible for lung adenocarcinoma cell behaviors induced by hsa_circ_0000326 in vitro. We introduced miR-338-3p mimics into hsa_circ_0000326-overexpressing cells and miR-338-3p inhibitors into hsa_circ_0000326-knockdown cells. According to the results of CCK-8 assays, silencing of hsa_circ_0000326 expression reduced the proliferation of A549 and H1299 cells, but miR-338-3p silencing attenuated this effect (Fig. 4a). Conversely, hsa_circ_0000326 significantly increased the proliferation of tumor cells, but miR-338-3p mimics reversed this effect (Fig. 4b). Additionally, reintroduction of miR-338-3p inhibitors attenuated the increase in apoptosis in hsa_circ_0000326-knockdown cells (Fig. 4c). Hsa_circ_0000326 overexpression decreased the apoptosis rate, and miR-338-3p mimics reversed this effect (Fig. 4d). The results of the transwell assays revealed that miR-338-3p silencing abolished the hsa_circ_0000326 knockdown-induced suppression of migration (Fig. 4e). However, hsa_circ_0000326 accelerated the migration of cells, while miR-338-3p clearly reversed this effect (Fig. 4f). Thus, hsa_circ_0000326 interacted with miR-338-3p to promote proliferation and migration and inhibit apoptosis in lung adenocarcinoma cells.

### Hsa_circ_0000326 inhibited the posttranscriptional activity of miR-338-3p and upregulated the expression of the miR-338-3p target RAB14

**Fig. 5:**
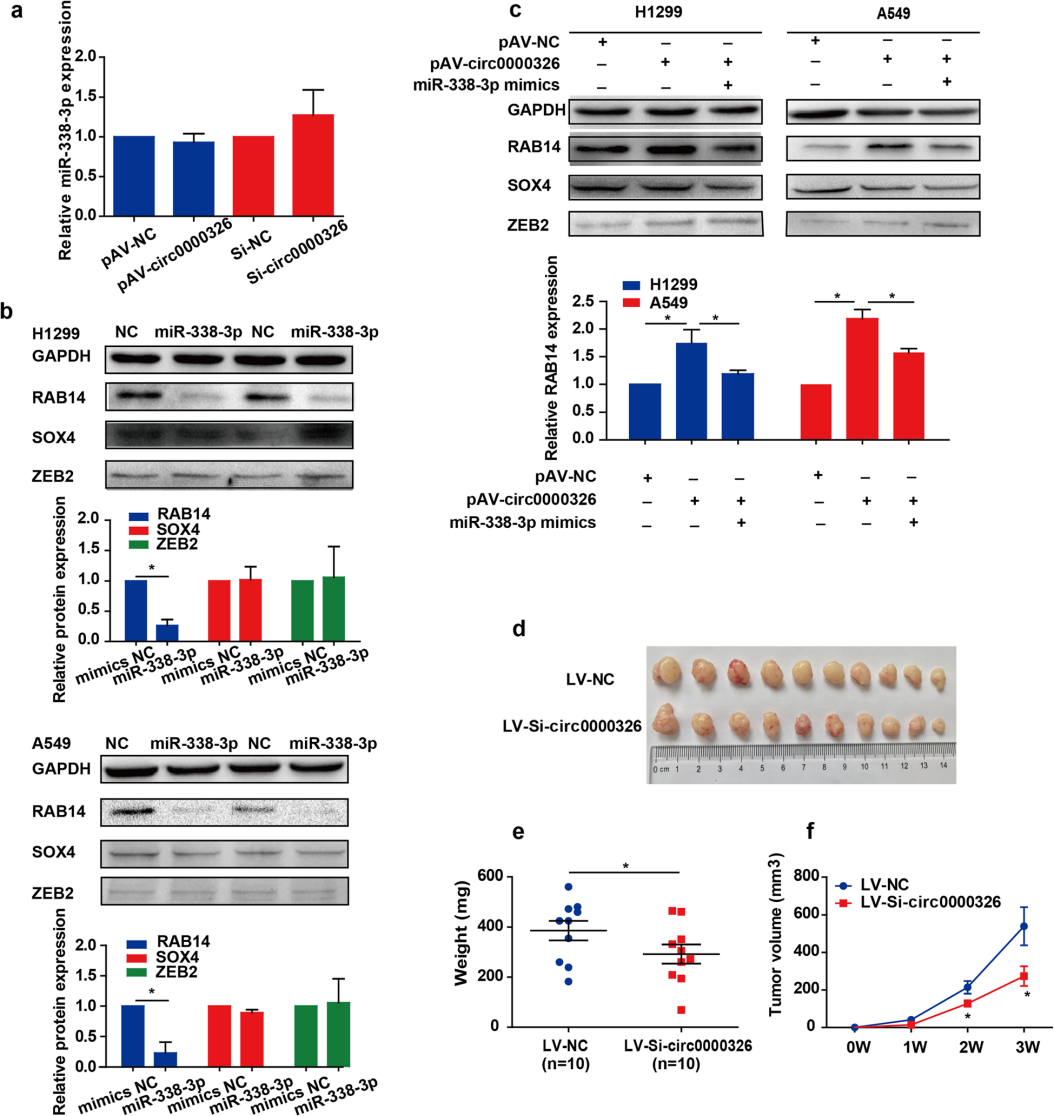
Hsa_circ_0000326 inhibited the posttranscriptional activity of miR-338-3p and upregulated the miR-338-3p target RAB14. (**a**) Expression of miR-338-3p in cells transfected with hsa_circ_0000326 overexpression plasmids or siRNAs. (**b**) Expression of SOX4, ZEB2 and RAB14 in miR-338-3p-expressing or miR-338-3p-depleted cells, as determined by western blot analysis. The expression levels were normalized to GAPDH expression levels. (**c**) Expression of SOX4, ZEB2 and RAB14 in cells transfected with the hsa_circ_0000326 overexpression plasmid (pAV-circ0000326) and miR-338-3p mimics. The bars with error bars represent the mean ± SD from three independent experiments. (**d**) Images of the xenograft tumors in each group 3 weeks after subcutaneous implantation of H1299 cells stably infected with hsa_circ_0000326-knockdown lentivirus (LV-si-circ0000326) or control lentivirus (LV-NC) (*n* = 7). (**e**) Effect of hsa_circ_0000326 knockdown on tumor weight 3 weeks after subcutaneous injection of H1299 cells stably infected with LV-si-circ0000326 or LV-NC. (**f**) Effect of intratumoral hsa_circ_0000326 knockdown on tumor growth in BALB/c nude mice. *P < 0.05

According to previous studies, circRNAs sponge miRNAs to terminate their regulatory effects on target genes [26]. Interestingly, hsa_circ_0000326 did not affect the expression of miR-338-3p (Fig. 5a), indicating that hsa_circ_0000326 regulated miR-338-3p at the posttranscriptional level. Because miRNAs might exert their tumor-suppressing or oncogenic functions by regulating the expression of target genes, three commonly used bioinformatics tools were applied to predict target genes. After considering the overlap among the genes predicted by TargetScan, PicTar and miRanda, RAB14, SOX4 and ZEB2 were selected as potential targets for further validation. The western blot results showed that introduction of exogenous miR-338-3p into A549 and H1299 cells reduced the levels of the RAB14 protein, indicating that RAB14, but not SOX4 or ZEB2, was the downstream target of miR-338-3p (Fig. 5b). Next, we explored whether hsa_circ_0000326 regulated the expression of RAB14. As shown in the western blots, hsa_circ_0000326 overexpression dramatically increased the levels of the RAB14 protein, and reintroduction of miR-338-3p reversed this effect (Fig. 5c).

### Silencing of hsa_circ_0000326 suppressed tumor growth

H1299 cells that were stably infected with LV-si-circ0000326 or LV-NC were subcutaneously injected into the right armpits of nude mice. Three weeks after injection, the tumors were removed and photographed (Fig. 5d). The weights of the xenograft tumors in which hsa_circ_0000326 expression was downregulated were significantly lower than those of the control xenograft tumors (Fig. 5e). In addition, tumors derived from circ0000326-knockdown clones grew at significantly slower rates than control tumors at all time points examined (Fig. 5f).

## Discussion

According to recent studies, circRNAs represent potentially novel and promising biomarkers in cancer and other diseases, such as hsa_circ_0000190 in gastric cancer [27], hsa_circRNA_103636 in major depressive disorder [28], and hsa_circ_0004277 in acute myeloid leukemia [29]. Functional and mechanistic studies have revealed that circRNAs exert regulatory effects on the initiation and progression of diseases [30, 31]. Notably, circRNAs have been shown to act as competing endogenous RNAs via the circRNA-miRNA-mRNA signaling axis, which participates in the initiation and progression of various cancers, such as colorectal cancer [32], gastric cancer [33], breast cancer [34], and esophageal cell carcinoma [35, 36].

Based on accumulating evidence, aberrant circRNA expression plays critical roles in lung cancer. Liu et al. reported the upregulation of certain circRNAs (circ-ZEB1.5, circ-ZEB1.19, circ-ZEB1.17 and circ-ZEB1.33) in normal lung tissue samples compared to lung cancer samples [37]. Yao et al. observed elevated expression of circRNA_100876 in NSCLC tissues compared to adjacent lung tissues by RT-PCR. Furthermore, Kaplan-Meier survival analysis showed that patients expressing high levels of circRNA_100876 had reduced overall survival times, and the levels of circRNA_100876 expression were found to be correlated with lymph node metastasis and NSCLC tumor stage [38]. Using microarray screening, Zhu et al. revealed that hsa_circ_0013958 expression is markedly upregulated in lung adenocarcinoma tissues. Hsa_circ_0013958 promotes cell proliferation and invasion and inhibits apoptosis by acting as a sponge of miR-134 and thus upregulating the expression of oncogenic cyclin D1 [39]. The classic circRNA ITCH has been found to be significantly downregulated in lung cancer tissues and to function as a sponge for miR-7 and miR-214, subsequently suppressing the activity of the Wnt/β-catenin signaling pathway [40]. According to microarray and RT-PCR data, hsa_circ_0043256 is upregulated in NSCLC cells in response to cinnamaldehyde treatment and might inhibit cell proliferation and induce apoptosis via the hsa_circ_0043256/miR-1252/ITCH axis in response to cinnamaldehyde [41].

In the present study, we used microarray technology to show that hsa_circ_0000326 expression was significantly upregulated in lung adenocarcinoma tissues, and we validated this result with a larger sample size using RT-PCR. Moreover, we found that high hsa_circ_0000326 expression correlated with T stage, N stage and tumor differentiation level. We then explored the biological role of hsa_circ_0000326 using gain- and loss-of-function assays. Hsa_circ_0000326 promoted cell proliferation by inhibiting apoptosis rather than by affecting the cell cycle and increased the migration of lung adenocarcinoma cells.

Based on emerging data, circRNAs exert key regulatory effects by acting as competing endogenous RNAs. According to bioinformatics analysis, miR-338-3p was predicted to interact with hsa_circ_0000326, and luciferase reporter assays further validated this interaction. We detected the subcellular localization of hsa_circ_0000326 and found that hsa_circ_0000326 was mainly located in the cytoplasm, the same subcellular location as miR-338-3p. Functional assays showed that miR-338-3p mimics reversed the effects of hsa_circ_0000326 overexpression and that miR-338-3p inhibition reversed the effects of hsa_circ_0000326 depletion. However, hsa_circ_0000326 did not affect miR-338-3p expression. In our opinion, hsa_circ_0000326 may competitively bind and inhibit the activity of miR-338-3p, rather than effect the expression. Just as previous study, CircNT5E [26] or hsa_circ_001569 [42] or Circular RNA-ZNF609 [43] or circRHOBTB3 [44] were identified as a sponge of miRNA and did not affect their expression. Therefore, by acting as a miR-338-3p sponge, hsa_circ_0000326 may competitively bind to and inhibit miR-338-3p activity, resulting in increased expression of miR-338-3p targets.

As shown in previous studies, miR-338-3p expression exhibits marked changes in various tumors, such as hepatocellular carcinoma [45], gastric cancer [46], and lung cancer [47]. In the present study, hsa_circ_0000326 functioned at the posttranscriptional level to inhibit the activity of miR-338-3p and prominently increased the proliferation and migration of lung adenocarcinoma cancer cells by acting as a miR-338-3p sponge. Consistent with the results of previous studies, miR-338-3p expression was significantly decreased and functioned as a tumor suppressor. Importantly, miR-338-3p inhibits proliferation and the epithelial-mesenchymal transition in vitro and suppresses tumor growth in vivo [48, 49].

Using three bioinformatic algorithms (TargetScan, PicTar and miRanda), we identified RAB14, SOX4 and ZEB2 as potential targets of miR-338-3p. RAB14 is a member of the RAB family of low-molecular-weight GTPases and is expressed in a wide range of cell lines [50]. As shown in the western blots, RAB14 was a target of miR-338-3p in lung adenocarcinoma cells, and hsa_circ_0000326 overexpression contributed to elevated levels of RAB14. Alterations in RAB14 expression have been documented in gastric cancer, renal cancer, nasopharyngeal carcinoma and lung cancer [47, 51, 52]. As an oncogene, RAB14 participates in many biological processes, such as cell proliferation, migration and invasion, by regulating the AKT signaling pathway [51, 53].

Finally, we established xenografts in nude mice to assess the function of hsa_circ_0000326 in vivo. Interestingly, hsa_circ_0000326 affected lung adenocarcinoma progression in vivo, as evidenced by the significantly lower weights and volumes LV-si-circ0000326 xenograft tumors than control xenograft tumors.

Our study had some limitations. First, due to the poor tumorigenesis of A549 cells in nude mice, we did not create stable knockdown hsa_circ_0000326 cell lines or subcutaneously inject mice with these cells. Second, the role of hsa_circ_0000326 on the tumor cell invasion was unknown, although we have conducted invasion assay and optimized the experimental conditions for many times. Third, A549 and H1299 were used as the experimental cells because of the significantly high expression of hsa_circ_0000326. Although the different expression pattern of P53 in A549 and H1299 cells and P53 acted as a tumor suppressor, we observed that hsa_circ_0000326 acted as a negative regulator of apoptosis, along with RAB14 overexpression in A549 and H1299 cells, which indicated P53 may not be the target for hsa_circ_0000326/miR-338-3p/RAB14 signal pathway. A recently study found RAB14 overexpression upregulated Bcl-2, an important anti-apoptotic protein thus inhibited apoptosis [54]. We hypothesis the underlying apoptosis mechanisms of hsa_circ_0000326 may be upregulated of Bcl-2 expression, which need to be further validated.

**Fig. 6:**
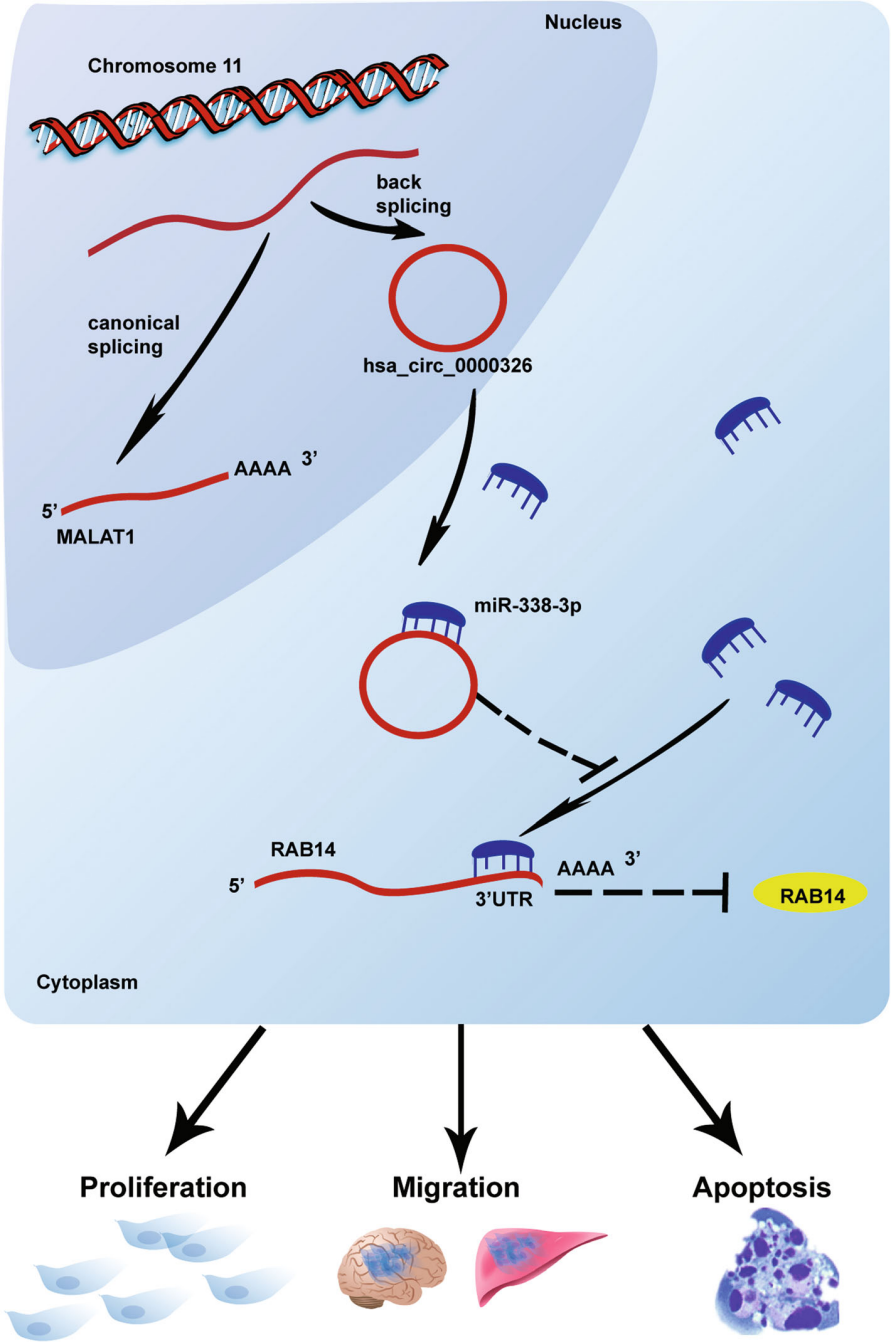
Schematic of the biological roles of hsa_circ_0000326 in lung adenocarcinoma carcinogenesis. Hsa_circ_0000326 could bind to miR-338-3p as a miRNA sponge, exerting its function via regulation of the downstream target RAB14. Knockdown of hsa_circ_0000326 could inhibit lung adenocarcinoma cell proliferation and migration and promote apoptosis in vitro and inhibit tumor formation in vivo

Aberrant hsa_circ_0000326 expression in lung adenocarcinoma promotes tumor proliferation and metastasis. We have constructed a model of the mechanism of hsa_circ_0000326 (Fig. 6) showing that the covalent binding of hsa_circ_0000326 to miR-338-3p increases RAB14 expression and participates in the progression of lung adenocarcinoma.

## Conclusions

Our study results reveal crucial roles for hsa_circ_0000326 in the proliferation, migration and apoptosis of lung adenocarcinoma cells and suggest that hsa_circ_0000326 may represent a potential therapeutic target in patients with lung adenocarcinoma.

## Supplementary information

**Additional file 1: Supplement fig. 1.** The expression of hsa_circ_0000326 had no effect on the cell cycle of A549. (a) Effect of hsa_circ_0000326 knockdown on cell cycle. (b) Effect of hsa_circ_0000326 overexpression on cell cycle, as determined by colony formation assay.

**Additional file 2: Supplement fig. 2.** RNAase H digestion results for hsa_circ_0000326.

**Additional file 3: Table S1.** The results of circRNAs array for lung adenocarcinoma tissues and adjacent normal tissues.

**Additional file 4: Table S2.** Dysregulated circRNAs in lung adenocarcinoma tissues compared with adjacent normal tissues.

**Additional file 5: Table S3.** Correlation between Circ_0000326 expression and clinical pathological characteristics.

## Data Availability

The datasets used and/or analysed during the current study are available from the corresponding author on reasonable request.

## Abbreviations

circRNAs: Circular RNAs
NSCLCs: Non-small cell lung cancers;
FISH: Fluorescence in situ hybridization
MALAT1: Metastasis associated lung adenocarcinoma transcript 1
SD: Standard deviation
RAB14: Ras-related protein Rab-14
SOX4: SRY-Box Transcription Factor 4
ZEB2: Zinc Finger E-Box Binding Homeobox 2
