# Supplementary figures and images for: Circular RNA hsa_circ_0000326 acts as a miR-338-3p sponge to facilitate lung adenocarcinoma progression
Source: J Exp Clin Cancer Res. 2020 Apr 5;39:57. doi: 10.1186/s13046-020-01556-4 (PMC7132982; doi:10.1186/s13046-020-01556-4)

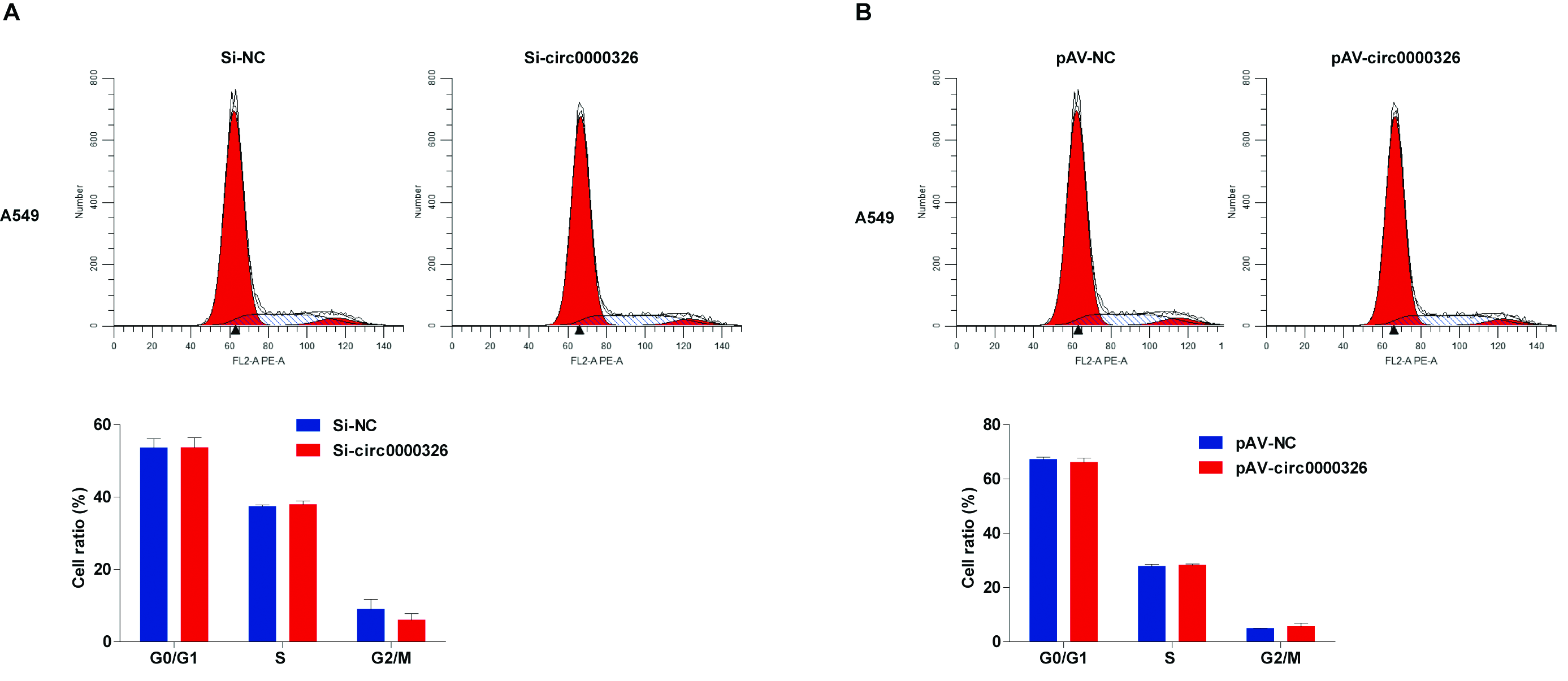

Supplement: Supplementary file 1 — Additional file 1: Supplement fig. 1. The expression of hsa_circ_0000326 had no effect on the cell cycle of A549. (a) Effect of hsa_circ_0000326 knockdown on cell cycle. (b) Effect of hsa_circ_0000326 overexpression on cell cycle, as determined by colony formation assay. [file 13046_2020_1556_MOESM1_ESM.tif]

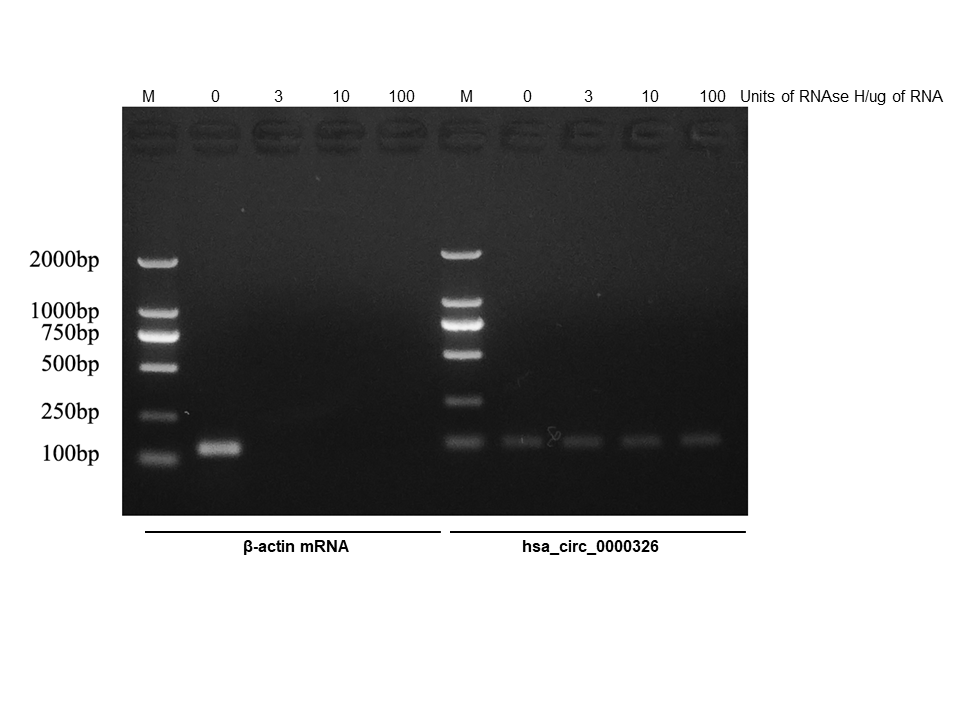

Supplement: Supplementary file 2 — Additional file 2: Supplement fig. 2. RNAase H digestion results for hsa_circ_0000326. [file 13046_2020_1556_MOESM2_ESM.tif]
