# Supplementary material for: Circular RNA hsa_circ_0000326 acts as a miR-338-3p sponge to facilitate lung adenocarcinoma progression
Source: J Exp Clin Cancer Res. 2020 Apr 5;39:57. doi: 10.1186/s13046-020-01556-4 (PMC7132982; doi:10.1186/s13046-020-01556-4)
Supplement: Supplementary file 4 — Additional file 4: Table S2. Dysregulated circRNAs in lung adenocarcinoma tissues compared with adjacent normal tissues. [file 13046_2020_1556_MOESM4_ESM.docx]

Table S2: Dysregulated circRNAs in lung adenocarcinoma tissues compared with adjacent normal tissues.

| Probe ID | CircRNA | Genesymbol | Chrom | Log_2_FC | Dysregulation |
| --- | --- | --- | --- | --- | --- |
| ASCRP3013462 | hsa_circ_0002082 | MALAT1 | chr11(+):65271199-65272066 | 5.44978 | up |
| ASCRP3009256 | hsa_circ_0000326 | MALAT1 | chr11(+):65272490-65272586 | 4.745248 | up |
| ASCRP3003345 | hsa_circ_0001588 | HIST1H4E | chr6(+):26204839-26205043 | 4.547723 | up |
| ASCRP3006491 | hsa_circ_0050898 | ACTN4 | chr19(+):39138266-39219794 | 4.157384 | up |
| ASCRP3007020 | hsa_circ_0000517 | RPPH1 | chr14(-):20811404-20811492 | 3.929398 | up |
| ASCRP3011389 | hsa_circ_0000518 | RPPH1 | chr14(-):20811404-20811554 | 3.927556 | up |
| ASCRP3013422 | hsa_circ_0032245 | FUT8 | chr14(+):65922338-65931191 | 3.682233 | up |
| ASCRP3007638 | hsa_circ_0087538 | FAM120A | chr9(+):96233422-96324586 | 3.57703 | up |
| ASCRP3005568 | hsa_circ_0006853 | RPPH1 | chr14(-):20811282-20811360 | 3.518585 | up |
| ASCRP3003030 | hsa_circ_0000514 | RPPH1 | chr14(-):20811305-20811436 | 3.494609 | up |
| ASCRP3010298 | hsa_circ_0005730 | [CDK7](http://www.ncbi.nlm.nih.gov/gene/?term=1022) | chr5(+):68553869-68558131 | -1.39843 | down |
| ASCRP3008433 | hsa_circ_0004943 | KIAA1549L | chr11(+):33563347-33566924 | -1.38478 | down |
| ASCRP3008573 | hsa_circ_0024524 | [VPS11](http://www.ncbi.nlm.nih.gov/gene/?term=55823) | chr11(+):118939940-118952024 | -1.36276 | down |
| ASCRP3008970 | hsa_circ_0081015 | [ANKIB1](http://www.ncbi.nlm.nih.gov/gene/?term=54467) | chr7(+):91974291-92000921 | -1.36181 | down |
| ASCRP3006838 | hsa_circ_0003997 | [CLMP](http://www.ncbi.nlm.nih.gov/gene/?term=79827) | chr11(-):122953792-122955421 | -1.33877 | down |
| ASCRP3007675 | hsa_circ_0048977 | [ZNF358](http://www.ncbi.nlm.nih.gov/gene/?term=140467) | chr19(+):7584090-7585911 | -1.33827 | down |
| ASCRP3008085 | hsa_circ_0045601 | [HN1](http://www.ncbi.nlm.nih.gov/gene/?term=51155) | chr17(-):73142760-73143748 | -1.33438 | down |
| ASCRP3008380 | hsa_circ_0053944 | [FAM98A](http://www.ncbi.nlm.nih.gov/gene/?term=25940) | chr2(-):33808728-33810511 | -1.33417 | down |
| ASCRP3010952 | hsa_circ_0046941 | [GNAL](http://www.ncbi.nlm.nih.gov/gene/?term=2774) | chr18(+):11752851-11753944 | -1.30984 | down |
| ASCRP3000519 | hsa_circ_0048410 | [LMNB2](http://www.ncbi.nlm.nih.gov/gene/?term=84823) | chr19(-):2428163-2431900 | -1.29701 | down |
