# Supplementary material for: Circular RNA hsa_circ_0000326 acts as a miR-338-3p sponge to facilitate lung adenocarcinoma progression
Source: J Exp Clin Cancer Res. 2020 Apr 5;39:57. doi: 10.1186/s13046-020-01556-4 (PMC7132982; doi:10.1186/s13046-020-01556-4)
Supplement: Supplementary file 5 — Additional file 5: Table S3. Correlation between Circ_0000326 expression and clinical pathological characteristics. [file 13046_2020_1556_MOESM5_ESM.docx]

Table S3: Correlation between Circ_0000326 expression and clinical pathological characteristics

| Circ_0000326 expression | | | |
| --- | --- | --- | --- |
| Characteristics | Low | High | P |
| Age | | | |
| ≤Mean(59) | 32 | 12 | 0.797 |
| ＞Mean(59) | 42 | 14 |  |
| Gender | | | |
| Male | 36 | 16 | 0.258 |
| Female | 38 | 10 |  |
| Smoking |  |  |  |
| Yes | 26 | 13 | 0.181 |
| No | 48 | 13 |  |
| Differentiation | | | |
| Well-moderate | 59 | 7 | 0.000* |
| Poor | 15 | 19 |  |
| T classification | | | |
| 1-2 | 66 | 19 | 0.048* |
| 3-4 | 8 | 7 |  |
| N classification | | | |
| 0-1 | 51 | 12 | 0.039* |
| 2-3 | 23 | 14 |  |
| Distant metastasis | | | |
| No | 66 | 22 | 0.537 |
| Yes | 8 | 4 |  |

*Significant association
